# NFAT1 and NFAT2 Differentially Regulate CTL Differentiation Upon Acute Viral Infection

**DOI:** 10.3389/fimmu.2019.00184

**Published:** 2019-02-15

**Authors:** Tianhao Xu, Ashleigh Keller, Gustavo J. Martinez

**Affiliations:** Department of Microbiology and Immunology, Chicago Medical School, Rosalind Franklin University, North Chicago, IL, United States

**Keywords:** NFAT1, NFAT2, CD8^+^ T cells, differentiation, effector, memory, LCMV

## Abstract

CD8^+^ T cell differentiation orchestrated by transcription regulators is critical for balancing pathogen eradication and long-term immunity by effector and memory CTLs, respectively. The transcription factor Nuclear Factor of Activated T cells (NFAT) family members are known for their roles in T cell development and activation but still largely undetermined in CD8^+^ T cell differentiation *in vivo*. Here, we interrogated the role of two NFAT family members, NFAT1 and NFAT2, in the effector and memory phase of CD8^+^ T cell differentiation using LCMV^Arm^ acute infection model. We found that NFAT1 is critical for effector population generation whereas NFAT2 is required for promoting memory CTLs in a cell intrinsic manner. Moreover, we found that mice lacking both NFAT1 and NFAT2 in T cells display a significant increase in KLRG1^hi^ CD127^hi^ population and are unable to clear an acute viral infection. NFAT-deficient CTLs showed different degrees of impaired IFN-γ and TNF-α expression with NFAT1 being mainly responsible for IFN-γ production upon *ex-vivo* stimulation as well as for antigen-specific cytotoxicity. Our results suggest that NFAT1 and NFAT2 have distinct roles in mediating CD8^+^ T cell differentiation and function.

## Introduction

CD8^+^ T cells are pivotal in combating intracellular pathogens and for tumor immune surveillance (1, 2). Upon recognizing their cognate antigen presented by antigen-presenting cells (APCs), naïve CD8^+^ T cells are activated, rapidly proliferate and differentiate into a heterogeneous pool of effector cells which display cytotoxic activity (3). The heterogeneity of activated CD8^+^ T cells has been characterized by the expression of different surface markers and transcription factors. Conventionally, effector cells are further categorized into two main subsets: short-lived effector T cells (SLECs) as KLRG1^hi^ CD127^lo^ and memory precursor effector cells (MPECs) as KLRG1^lo^ CD127^hi^ (4). Upon clearance of the pathogen or tumor, most antigen-specific SLECs die due to lack of antigen stimulation. The remaining MPECs are responsible for providing long-term protection upon subsequent reinfections (4, 5). A recent study shows that SLECs could also contribute to part of the memory population pool, demonstrating CTLs' plasticity (6).

The dynamic CD8^+^ T cell terminal effector and memory differentiation balance and heterogeneity is orchestrated by transcription regulators, many of which are still unidentified. The transcription factors Tbet and Eomesodermin (Eomes) have long been known to directly control CD8^+^ T cell effector and memory differentiation, respectively (7, 8). In recent years, other transcription factors have been identified to regulate CTL differentiation: Id2 and Blimp-1 are critical for effector CTL formation while Id3, Bcl-6, Tcf-1, and Runx3 are cardinal for memory CD8^+^ T cell development (8–12). The b-zip transcription factor BATF is required for early TCR signaling amplification and partnering with other transcription factors, such as JUN and IRF4 (13–16). Runx3, a transcription factor responsible for mediating CD8^+^ T cell commitment in the thymus by antagonizing ThPok, which drives CD4^+^ Th cell fate (17), has been also recently implicated in the early TCR signaling events during priming to promote the accessibility of chromatin containing IRF, bZIP, and PRDM1 binding motifs resulting in the strengthening of memory population differentiation (18). Despite the knowledge on how these transcription factors regulate CTL differentiation *in vivo*, the exact mechanisms that set off the effector and memory CD8^+^ T cells development are still unclear, especially those transcription factors downstream of early TCR signaling events.

Nuclear factor of activated T cells (NFAT) is a well-known transcriptional regulator of T cell activation and plays an essential role in T cell development, activation and function. There are five NFAT family members, among them, NFAT1, NFAT2, and NFAT4 are expressed in T cells downstream of Ca^2+^-Calcineurin signaling (19). All NFAT members share a consensus DNA binding motif which allows them to bind to the same DNA sequence (20–22). Upon T cell activation, NFAT is dephosphorylated and rapidly translocates into the nucleus to regulate gene expression via cooperation with other transcriptional regulators (21, 23). Recent studies have shown that different NFAT members have distinct properties and roles in CD4^+^ T lymphocytes, especially in Th1, Th17, Tfh, and Tregs (24–26). *In vitro* study of CD8^+^ T cells selectively deficient in NFAT1, NFAT2, or NFAT4 and/or combination demonstrated a distinct role of these transcription factors regulating cytokines and inhibitory receptors expression (27). However, the distinction among NFAT members establishing CD8^+^ T cell effector and memory differentiation *in vivo* is still undetermined.

In this study, we examined the role of NFAT1 and NFAT2 in CTL differentiation and function using an acute lymphocytic choriomeningitis virus Armstrong strain (LCMV^Arm^) infection model (28, 29). We characterized LCMV-specific CD8^+^ T cell effector and memory population in mice deficient in NFAT1, mice with T cell-specific NFAT2 deficiency or with double deficiency of NFAT1 and NFAT2 in T cells. We found that NFAT1 is required for effector while NFAT2 is necessary for memory population generation. Mice deficient in both NFAT1 and NFAT2 have delayed memory differentiation and are unable to control an acute viral infection. Moreover, we also observed reduced cytokine production in all NFAT-deficient cells, with cells deficient in both transcription factors having the strongest effect, as well as an imbalanced Tbet and Eomes expression. The defect in CTL differentiation was cell-intrinsic, as evidenced by both mixed bone marrow chimera experiments and adoptive transfer of NFAT-deficient antigen-specific P14 TCR transgenic cells. These results suggest that NFAT1 and NFAT2 are indispensable and have distinct roles in initiating CD8^+^ T cell effector and memory differentiation and function.

## Materials and Methods

### Mice

All mice from C57BL/6 background used in the experiments were 6–8 weeks old, sex, and age matched. NFAT1^−/−^ and NFAT2^fl/fl^ CD4-Cre and NFAT1^−/−^ NFAT2^fl/fl^ CD4-Cre mice were obtained from La Jolla Institute for Allergy and Immunology (LJI, San Diego, CA) and have been described (24). NFAT1^−/−^ mice were crossed with NFAT2^fl/fl^ CD4-Cre^+^ to generate NFAT1^−/−^ NFAT2^fl/fl^ CD4-Cre^+^ (NFAT1/2 DKO) mice. P14 Thy1.1 or P14 TCRα^−/−^ TCR transgenic mice were further crossed with NFAT deficient mice described above. For the mixed bone marrow chimera experiment, bone marrow cells were isolated from tibia and femur from B6.SJL CD45.1 mice, and mixed 1:1 ratio with bone marrow cells from C57BL/6 CD45.2 WT, NFAT1^−/−^, NFAT2^fl/fl^ CD4-Cre^+^, and NFAT1^−/−^ NFAT2^fl/fl^ CD4-Cre^+^ mice. Then, 7 million mixed bone marrow cells were transferred into lethally irradiated recipient B6SJL mice. All mice were maintained in specific-pathogen-free barrier facilities and used according to protocols approved by the Rosalind Franklin University of Medicine and Science Institutional Animal Care and Use Committee (IACUC).

### Lymphocytic Choriomeningitis Virus (LCMV) Models

WT, NFAT1^−/−^ (NFAT1 KO), NFAT2^fl/fl^ CD4Cre^+^ (NFAT2 TKO), or NFAT1^−/−^, NFAT2^fl/fl^ CD4Cre^+^ (NFAT1/2 DKO), as well as mixed bone marrow chimera mice were infected intraperitoneally (i.p) with 2 × 10^5^ PFU of LCMV Armstrong (LCMV^Arm^) kindly provided by Dr. Shane Crotty at LJI. After infection, splenocytes, and serum were harvested. Serum viral titers were measured by plaque assay as described (29).

### Cell Staining and Flow Cytometry

Single cell suspension isolated from spleens or heparinized blood were treated with RBC lysis buffer, washed and incubated with tetramer and antibody cocktails for surface staining. Single cell suspensions were initially incubated with LCMV tetramers H2D^b^-GP33-41 (KAVYNFATC) Alexa647, H2D^b^-GP276-286 (SGVENPGGYCL) BV421, and H2D^b^-NP396-404 (FQPQNGQFI) PE kindly obtained from the NIH Tetramer Facility, followed by staining of cell surface molecules including CD44, CD4, B220, CD8, KLRG1, CD127, and CXCR3. For intracellular transcription factor and cytokine staining, cells were then fixed, permeabilized and stained with antibody against Tbet, Eomes, IFN-γ, TNF-α, using eBioscience intracellular staining kits. Expression of these markers was assessed by flow cytometry using BD LSRII. The antibodies and reagents used are listed in Supplementary Table 1.

### T Cells Isolation, Culture, Cytokine Production, and Cytotoxicity Assay

Spleen and lymph nodes were harvested, naïve CD8^+^ cells were purified using Stem Cell EasySep kit from pooled spleen and lymph node cells. Dulbecco's modified Eagle's medium (DMEM) supplemented with 10% heat-inactivated fetal bovine serum, 2 mM L-glutamine, penicillin-streptomycin, non-essential amino acids, sodium pyruvate, vitamins, 10 mM HEPES, and 50 uM 2-mercaptoethanol were used for T cell culture (24). Cells (10^6^ cells/ml) were stimulated with anti-CD3 (clone3 2C11) and anti-CD28 (clone 37.51) (1 μg/ml each, both from BioXcell), 2U IL-2 and 50 ng/ml gentamycin in 6-well plates that had been pre-coated with 50 μg/ml goat anti-hamster IgG (Pierce, Life Technologies). On day 2, cells were removed from the initial stimulus, and cultured at 0.5 million cells/ml with 10U/ml of recombinant human IL-2 (30).

To assess cytokine production and the cytotoxicity activity, on day 6 after activation, cells were co-cultured at different ratios with GFP-expressing parental mammary carcinoma cell line EO771 (negative control to determine non-specific target lysis), or EO771 cells expressing the cognate antigen GP33-41 (kindly provided by Mark Sundrud at TSRI-FL). After 12 h incubation, remaining live GFP-expressing EO771 cells were determined by FACS as a measurement of cytotoxic activity. GP33-41-expressing EO771 cells cultured in the absence of CTL were used as baseline for cell death. Cytokine production was also measured upon restimulation with PMA and Ionomycin or with RAG1^−/−^ splenocytes incubated with 0.2 **μ**g/ml of GP33-41 peptide for 4 h in the presence of BrefeldinA.

### Quantitative Real-Time RT-PCR

Total RNA was prepared from T cells after stimulation using TRIzol reagent (Invitrogen). cDNA was synthesized using Superscript reverse transcriptase and oligo(dT) primers (Invitrogen), and gene expression was examined with 7900 Real Time PCR System (Applied Biosystems) using Power SYBR green PCR Master Mix (Thermo Fisher). Gene expression was normalized to *Rpl32* (encodes L32 ribosomal protein) gene expression. The following primers were utilized: *Rpl32* forward: 5′-CGTCTCAGGCCTTCAGTGAG-3′; *Rpl32* reverse*:* 5′-CAAGAGGGAGAGCAAGCCTA-3′; *Ifng* forward: 5′-ATCTGGAGGAACTGGCAAAA-3′; *Ifng* reverse: 5′-TTCAAGACTTCAAAGAGTCTGAGGTA-3′; *Il2* forward: 5′-TTGTGCTCCTTGTCAACAGC-3′; *Il2* reverse: 5′-CTGGGGAGTTTCAGGTTCCT-3′; *Tnf* forward: 5′-GCCTCTTCTCATTCCTGCTTG-3′; *Tnf* reverse: 5′-CTGATGAGAGGGAGGCCATT-3′; *Gzmb* forward: 5′-CCACTCTCGACCCTACATGG-3′; *Gzmb* reverse: 5′-GGCCCCCAAAGTGACATTTATT-3′; *Prf1* forward: 5′-AATATCAATAACGACTGGCGTGT-3′; *Prf1* reverse: 5′-CATGTTTGCCTCTGGCCTA-3′.

### Statistics and Analysis

Flow cytometry data was analyzed with FlowJo (Version 9.9.4). Graphs are plotted using Prism 7 graph pad. Statistical analysis was performed using non-paired One-Way ANOVA followed by Tukey's multiple comparisons, unpaired two-tailed *t*-test or Two-Way ANOVA followed by Dunnett comparisons. Correlation test was done using the non-parametric Spearman correlation coefficient. ^*^p ≤ 0.05, ^**^p ≤ 0.01, ^***^p ≤ 0.001, ^****^p ≤ 0.0001.

## Results

### NFAT1 and NFAT2 Distinctively Regulate CD8^+^ T Cell Effector and Memory Differentiation During Acute LCMV^Arm^ Infection

**Figure 1:**
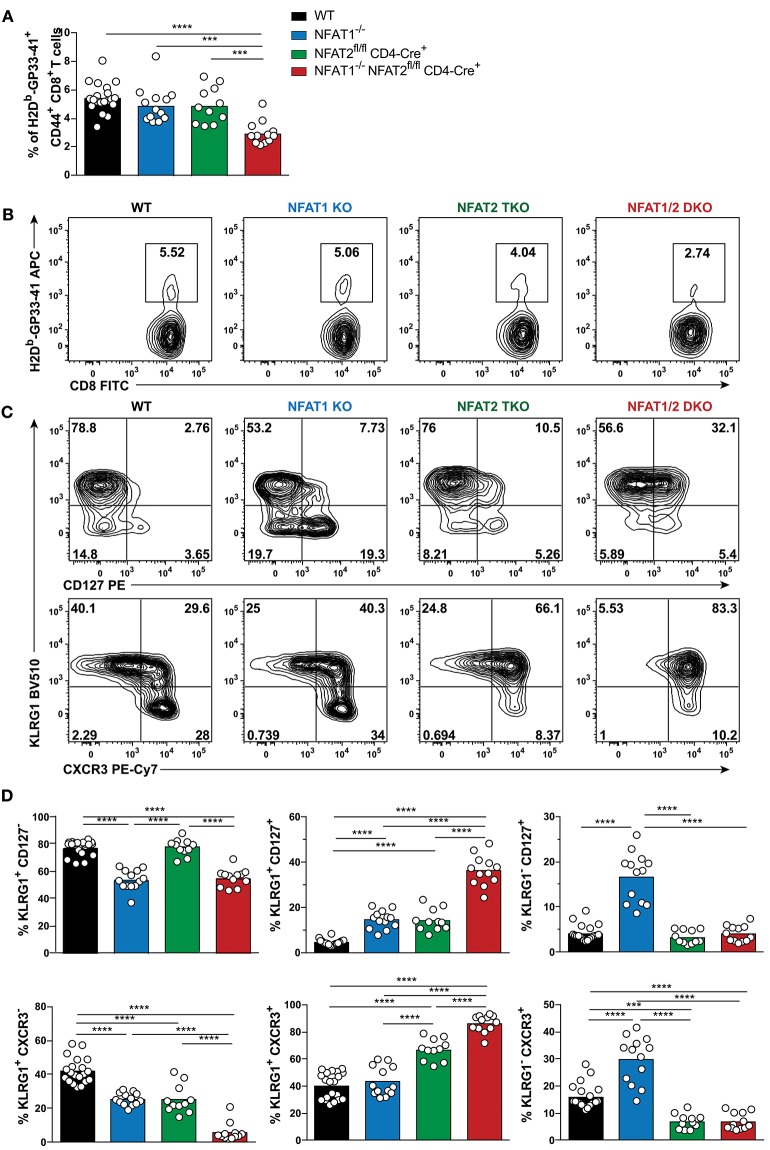
NFAT1 and NFAT2 distinctively regulate CD8^+^ T cell effector and memory differentiation during acute LCMV^Arm^ infection. Mice were infected with LCMV Arm (2 × 10^5^ PFU/mouse) intraperitoneally (i.p.). Spleens were harvested on day 8 after infection, and phenotypic characterization of CD8^+^ T cells was performed. **(A,B)** Frequency of H2D^b^-gp33-41^+^ (LCMV-specific) cells gated on CD8^+^ CD44^hi^ CD4^−^ B220^−^ splenocytes were determined, and presented as the combined data from three independent experiments **(A)** or a representative contour plot **(B)**. **(C,D)** Expression of KLRG1, CD127, and CXCR3 gated on H2D^b^-gp33-41^+^ CD8^+^ CD44^hi^ CD4^−^ B220^−^ cells was determined as a representative contour plot **(C)** and pooled data from three independent experiments **(D)**. **(A,D)** Statistical analysis was performed on three independent experiments combined using non-paired One-Way ANOVA followed by Tukey's multiple comparisons. ^*^p ≤ 0.05, ^**^p ≤ 0.01, ^***^p ≤ 0.001, ^****^p ≤ 0.0001.

To examine the role of NFAT1 and NFAT2 in CD8^+^ T cell differentiation during acute viral infection, we used mice deficient in NFAT1 (NFAT1^−/−^, referred as NFAT1 KO) and mice with conditional T cell-specific deficiency of NFAT2 (NFAT2^fl/fl^ CD4-Cre^+^, referred herein as NFAT2 TKO). We bred NFAT1KO mice with NFAT2 TKO mice to generate NFAT1^−/−^ NFAT2^fl/fl^ CD4-Cre^+^ (hereafter mentioned as NFAT1/2 DKO) mice. Six to eight weeks old WT, NFAT1 KO, NFAT2 TKO, and NFAT1/2 DKO mice were intraperitoneally injected with LCMV^Arm^. Eight days post-infection, corresponding to the peak of the T cell effector response, we characterized the generation of CD8^+^ T cell effector and memory antigen-specific populations using LCMV H2D^b^-GP33-41 tetramer staining (Figures 1A,B, S1). We found that NFAT1 KO mice have increased spleen cellularity, whereas NFAT2 TKO and NFAT1/2 DKO mice have significant fewer splenocytes compared to WT controls (Figure S1B). NFAT1/2 DKO mice also displayed significantly lower percentage and number of antigen-specific CD8^+^ T cells (LCMV tetramer H2D^b^-GP33-41^+^) compared to their WT counterparts (Figures 1A,B, S1E). Moreover, NFAT2 TKO and NFAT1/2 DKO mice exhibit lower number of CD8^+^ T cells in spleen (Figures S1C,D).

To evaluate the effect of deficiency in distinct NFAT members in CTL differentiation, we determined the SLECs, MPECs and effector memory (KLRG1^hi^ CD127^hi^) populations on antigen-specific CD8^+^ T cells recognizing GP33-41 peptide of LCMV. NFAT1 KO mice exhibit a significantly decreased SLEC population and an increase in CD127^hi^ cells, with over 3-fold increase in effector memory and MPEC populations compared to WT controls (Figures 1C,D). On the contrary, NFAT2 TKO mice displayed an increase in KLRG1-expressing cells, particularly the effector memory subset, while NFAT1/2 DKO mice had a significant increase in effector memory cells expressing both KLRG1 and CD127, suggesting an additive effect of NFAT1 and NFAT2 deficiency. To exclude that the phenotype observed was specific only to LCMV H2D^b^-GP33-41 tetramer, we additionally evaluated the CTL differentiation in H2D^b^-GP276-286 and H2D^b^-NP396-404 specific CD8^+^ T cells (Figures S1F,G). We observed that each and combined NFAT deficiency led to an altered effector/memory CTL differentiation as described above irrespective of the antigen specificity.

The expression of the chemokine receptor CXCR3 is upregulated during CD8^+^ T cell activation, and maintained through the transition from effector to memory population, though it remains at higher levels in memory CD27^hi^ CTLs (31–33). CXCR3 is important for trafficking of CTLs to peripheral tissues and lymphoid compartments allowing for the interaction with antigen-presenting cells, and further sustaining a type-1 inflammatory response (32). Using this marker, we also found a differential generation of KLRG1^lo^ CXCR3^hi^ and KLRG1^hi^ CXCR3^hi^ populations upon NFAT1 or NFAT2 deficiency (Figures 1C,D). Moreover, NFAT1/2 DKO mice have more than eighty percent of the population skewed toward KLRG1^hi^ CXCR3^hi^. Our results indicate that at the peak of the immune response in LCMV^Arm^ acute infection model, deficiency in NFAT1 or NFAT2 results in distinct CD8^+^ T cell differentiation outcomes: NFAT1 controls proper effector cell generation while NFAT2 regulates memory population formation.

**Figure 2:**
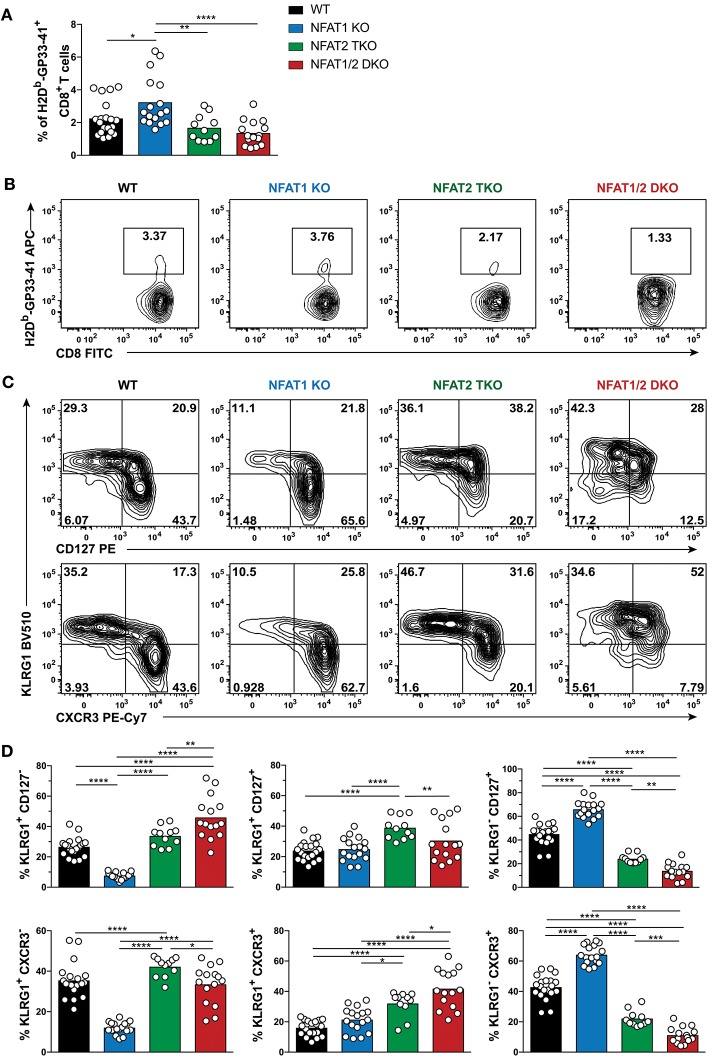
Dysregulated CD8^+^ T cell differentiation in NFAT-deficient mice is sustained during memory response *in vivo*. Mice were infected with LCMV Arm (2 × 10^5^ PFU/mouse) i.p. Spleens were harvested 30 days post-infection and CD8^+^ T cells were phenotypically characterized. **(A,B)** Frequency of H2D^b^-gp33-41^+^ (LCMV-specific) cells analyzed on CD8^+^ CD4^−^ B220^−^ cells. A representative contour plot is shown in **(B)** and the combined data from three independent experiments depicted in **(A)**. **(C,D)** Expression of KLRG1, CD127, and CXCR3 on CD8^+^ CD4^−^ B220^−^ H2D^b^-gp33-41^+^ cells shown as a representative contour plot **(C)** and pooled data from three independent studies **(D)**. Statistical analysis was done using non-paired One-Way ANOVA followed by Tukey's multiple comparisons. ^*^p ≤ 0.05, ^**^p ≤ 0.01, ^***^p ≤ 0.001, ^****^p ≤ 0.0001.

To determine if the phenotype observed in the different NFAT deficient mice is sustained to a memory time point, we analyzed effector and memory population in WT, NFAT1 KO, NFAT2 TKO and NFAT1/2 DKO mice 30 days p.i. We found a higher percentage of H2D^b^-GP33-41^+^ CD8^+^ T cells in NFAT1 KO mice while NFAT1/2 DKO mice showed a trend of reduced frequency and significant decreased total numbers of H2D^b^-GP33-41 specific CTLs compared to WT controls (Figures 2A,B, S2B–D). As expected, in WT mice, over 60% of the WT H2D^b^-GP33-41 specific CD8^+^ T cells express the memory marker CD127, of which only around 25% retain KLRG1 expression suggesting the formation of a stable memory population (Figures 2C,D). Consistent with what we observed at the peak of the immune response on day 8, we found a significant further expansion of the memory population in NFAT1 KO mice on day 30 post-infection. Similarly, NFAT2 TKO mice displayed a reduction in memory (CD127^hi^ KRLG1^lo^) cells, and an overall increase in KLRG1-expressing cells, particularly KLRG1^hi^ CD127^hi^ subset, compared to WT controls (Figures 2C,D). Notably, in NFAT1/2 DKO mice, we detected a reduction of CD127 single-positive and an increase in KLRG1 single-positive cells compared to other groups, suggesting a delayed transition to a memory phenotype. Similar phenotypic changes were also observed in the expression of CXCR3 (Figures 2C,D). Altogether, NFAT1 and NFAT2 differentially promote CD8^+^ T cell differentiation into effector and memory population, respectively, and double deficiency resulted in a reduced antigen-specific CTL population. Our results suggest that NFAT1 and NFAT2 not only play a role in T cell activation but are also crucial for driving proper CD8^+^ T cell commitment potential.

### Cell-Intrinsic Effect in the Generation of Effector And Memory Responses Mediated by NFAT Family Members

**Figure 3:**
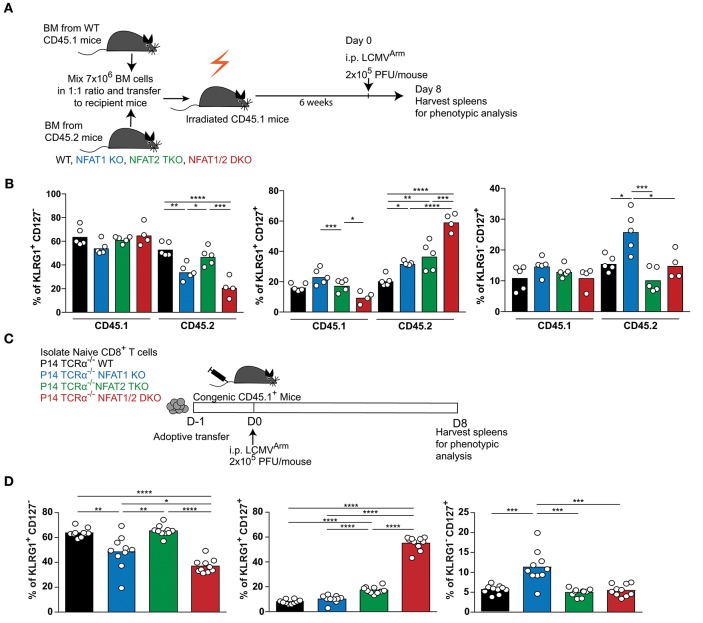
Cell-intrinsic regulation of CD8^+^ T cell differentiation by NFAT1 and NFAT2. **(A,B)** Mixed bone marrow chimera mice were generated by transferring into lethally irradiated CD45.1 mice a mix (1:1 ratio) of CD45.1 WT bone marrow cells with CD45.2 WT or NFAT-deficient bone marrow cells. Six weeks after reconstitution, mice were infected with LCMV Arm (2 × 10^5^ PFU/mouse) i.p. Spleens were harvested on day 8 after infection, and phenotypic characterization of CD8^+^ T cells was performed. **(A)** Schematic representation of the experimental design. **(B)** Expression of KLRG1 and CD127 was determined on antigen-specific (H2D^b^-gp33-41^+^) CD8^+^ T cells in CD45.1 and CD45.2 compartments. A representative experiment out of two is shown. **(C,D)** P14 TCR transgenic *Tcra*^−/−^ CD8^+^ T cells from WT or NFAT-deficient mice were adoptively transferred into CD45.1 congenic mice. One day later, mice were infected with LCMV Arm (2 × 10^5^ PFU/mouse) i.p. Spleens were harvested on day 8 after infection, and phenotypic characterization of adoptively transferred CD8^+^ T cells was performed. **(C)** Schematic representation of experimental design. **(D)** Expression of KLRG1 and CD127 was determined on adoptively transferred P14 cells as combined data from two independent experiments. Statistical analysis was done using non-paired One-Way ANOVA followed by Tukey's multiple comparisons **(B)** or student *T*-test **(D)**. ^*^p ≤ 0.05, ^**^p ≤ 0.01, ^***^p ≤ 0.001, ^****^p ≤ 0.0001.

Given that NFAT1 germline KO mice, NFAT2 T cell knockout mice and NFAT1/2 DKO mice were used, the differences in the effector and memory CTL differentiation could be due to cell intrinsic or extrinsic effects. To address this, we performed a mixed bone marrow chimera transfer experiment (Figures 3A,B, S3A–D). WT bone marrow cells carrying CD45.1 marker were mixed in a 1:1 ratio with WT, NFAT1 KO, NFAT2 TKO, or NFAT1/2 DKO bone marrow cells carrying CD45.2 marker, and co-transferred to lethally irradiated CD45.1 recipient mice. After 6 weeks, bone marrow reconstituted mice were infected with LCMV^Arm^ and analyzed for the generation of effector and memory populations on day 8 post-infection. While there are no differences in total splenocytes or the percentage of H2D^b^-GP33-41 specific CD45.1 CTLs among the different groups (Figures S3A,B), mice receiving NFAT1/2 DKO bone marrows showed a trend of decreased H2D^b^-GP33-41 specific CTLs in the CD45.2 compartment compared to the control cells, similar to what we have observed in the germline KO mice. We analyzed the effector and memory CD8^+^ T cells differentiation by measuring KLRG1 and CD127 expression in both CD45.1 and CD45.2 population (Figures 3B, S3D). We found that cells lacking NFAT1 were skewed toward a memory CTL population, cells lacking NFAT2 showed higher effector memory cells, and cells with NFAT1/2 double deficiency displayed an additive effect (Figures 3B, S3D). In addition, we adoptively transferred NFAT-deficient naïve P14 TCRα^−/−^ CTLs into congenic mice followed by LCMV^Arm^ infection (Figures 3C,D, S3E,F). Similar altered effector and memory differentiation was found upon NFAT deficiency (Figures 3C,D, S3E,F). Thus, these results indicate the NFAT1 and NFAT2 regulate CTL differentiation in a cell intrinsic manner.

### NFAT1 Deficiency Results in Reduced Effector Function, Which Is Further Compromised Upon Compound NFAT2 Deficiency

**Figure 4:**
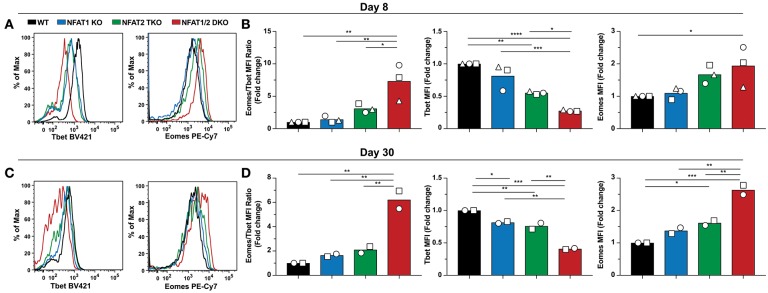
NFAT1 and NFAT2 regulate Tbet and Eomes expression. Expression of Tbet and Eomes was determined on antigen-specific cells from day 8 and 30 experiments depicted in Figures 1, 2. A representative histogram showing Tbet and Eomes MFI is shown on H2D^b^-gp33-41^+^ CD8^+^ T cells from day 8-infected mice **(A)** and day 30-infected mice **(C)**. Eomes and Tbet MFI fold change ratio as well as the fold change relative to WT mice was calculated by comparing the average Eomes or Tbet MFI fold change from different genotypes in each individual experiment on day8 **(B)** and day 30 **(D)**. Statistical analysis was done using non-paired One-Way ANOVA followed by Tukey's multiple comparisons. ^*^p ≤ 0.05, ^**^p ≤ 0.01, ^***^p ≤ 0.001, ^****^p ≤ 0.0001.

To assess if the abnormal CD8^+^ T cell differentiation upon deficiency of distinct NFAT family members also results in further transcriptional and functional changes, we initially determined the expression of two transcription factors Tbet and Eomes, which have been referred to as master regulators of CTL differentiation controlling the generation of effector and memory cells, respectively (34–36), on both day 8 and 30 post-LCMV infection (Figure 4). We found that Tbet expression was significantly reduced in NFAT2 TKO as well as NFAT1/2 DKO mice, with a slight, but not statistically different decrease in NFAT1 KO mice on day 8 p.i (Figures 4A,B). On the contrary, Eomes expression was significantly increased in NFAT1/2 DKO, and showed a consistent trend toward an increase, although not statistically significant, in NFAT2 TKO CD8^+^ T cells compared to WT controls. To delineate the relative expression between these two transcription factors, we calculated the fold change of Eomes vs. Tbet expression (using MFI) relative to WT cells (Figure 4B). CTLs lacking NFAT2 showed a slight increase in the Eomes:Tbet ratio, while cells with NFAT1 and NFAT2 double deficiency displayed an average of more than 5-fold increase in Eomes:Tbet compared to WT controls (Figure 4B). This dysregulated Eomes:Tbet expression was observed irrespective of the subpopulation analyzed (effector or memory cells based on KLRG1 and CD127 expression), and thus is not due to differences in frequencies of effector or memory CTLs (data not shown). Similar changes in Eomes:Tbet ratio were observed on day 30, a time point in which Tbet expression is reduced due to a memory transition (Figures 4C,D). The altered Tbet vs. Eomes ratio in NFAT1/2 DKO mice was a result of not only diminished Tbet but also increased Eomes expression (Figures 4B,D). However, at this time point, single NFAT deficiency did not show significant differences in expression of these two transcription factors (Figure 4D). Our results suggest that NFAT transcription factors regulate expression of Tbet and Eomes *in vivo*. This is in fact consistent with the presence of direct NFAT1, and to a lesser extent NFAT2, binding in both *Tbx21* and *Eomes* loci as shown using previously published ChIP-seq data (27, 37) (Figure S4).

**Figure 5:**
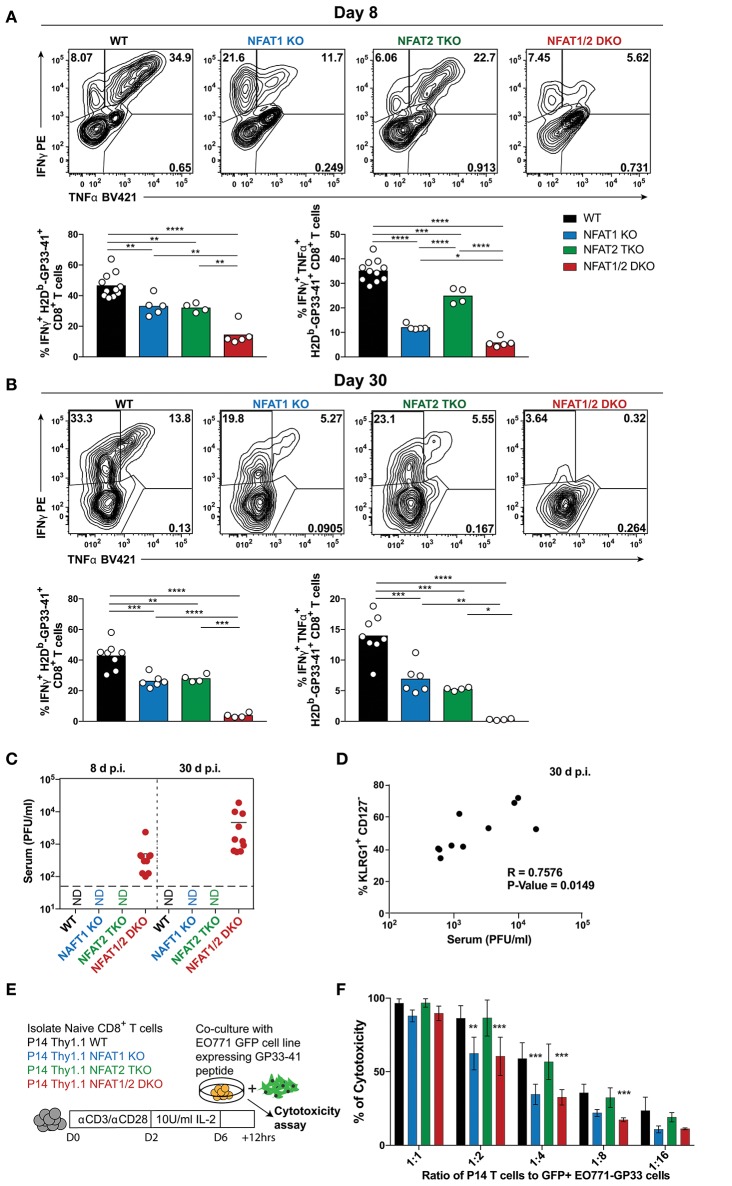
NFAT1 and NFAT2 differentially contribute to CTLs' effector function. **(A,B)** Cytokine production upon re-stimulation of total splenocytes was determined on antigen-specific cells from day 8 and 30 experiments depicted in Figures 1, 2. Expression of IFNγ and TNFα is shown in H2D^b^-gp33-41^+^ CD8^+^ T cells from day 8 **(A)** and day 30 **(B)** infections analyzed by One-Way ANOVA. **(C)** Viral titers from serum of each individual mouse collected from day 8 and 30 experiments (Figures 1, 2) was determined by plaque assay as Plaque Forming Units (PFU) per ml of serum. N.D., not detectable. **(D)** Spearman non-parametric correlation between percentage of KLRG1^+^ CD127^−^ antigen-specific cells from NFAT1/2 DKO mice 30d post-infection, and viral titers in serum (PFU/ml) (*p*-Value = 0.0149, *R* = 0.7576). **(E,F)** *In vitro*-cultured memory-like P14 TCR transgenic T cells were incubated with EO771-GFP gp33-41^+^ target cells in series dilution for 12 h. The percentage of GFP^+^ tumor cells was determined, and the percentage of cytotoxicity calculated based on CD8^−^ GFP^−^ cells. The data shows the mean +/− SD from three independent experiments, and was analyzed by Two-Way ANOVA followed by Dunnett comparisons ^*^p ≤ 0.05, ^**^p ≤ 0.01, ^***^p ≤ 0.001, ^****^p ≤ 0.0001.

To determine the impact of NFAT deficiency on CTL function, we initially determined cytokine production in antigen-specific CD8^+^ T cells after *ex vivo* stimulation with PMA and ionomycin (Figures 5A,B). The percentage of CTLs producing both IFN-γ and TNF-α were reduced in all NFAT-deficient cells, with NFAT1/2 double deficient cells displaying almost complete lack of cytokine production compared to WT controls both on day 8 and 30 (Figures 5A,B). Given that NFAT deficiency results in defective cytokine production, and Eomes levels are increased, which is also characteristic of exhausted cells (38), we hypothesized that these mice were unable to properly control the viral infection. For this purpose, we determined serum viral load in our experimental animals both on day 8 and 30 (Figure 5C). We observed that NFAT1/2 DKO mice were unable to control acute LCMV infection and the viral load increased over time, suggesting that NFATs are crucial for eliciting a proper immune response preventing viral persistence. This is consistent with a previous publication in which mice lacking both STIM1 and STIM2, which are upstream of NFAT signaling, were reported to have viral reoccurrence in the serum upon acute LCMV infection (39). Thus, our results pinpoint NFATs, within the calcium signaling pathways, as vital regulators of CTL function. Moreover, we observed a positive correlation between the viral load and the percentage of KLRG1^hi^ CD127^lo^ population suggesting that a delayed transition to a memory phenotype is associated with viral persistence (Figure 5D).

To further evaluate the role of NFAT members in CTL function, we utilized an *in vitro* antigen-specific cytotoxicity assay (Figures 5E,F, S5A–C). For this purpose, WT or NFAT-deficient P14^+^ TCR transgenic naïve T cells were activated and differentiated into memory-like CTLs, as previously described (30). These cells were then incubated at different ratios with parental breast cancer cell line EO771 expressing GFP (as negative controls) or EO771-GFP cells expressing GP33-41 (target cells) for 12 h. Cytotoxicity was determined as the frequency of CD8^−^ GFP^−^ cells. We did not observe any significant differences in antigen-specific killing between NFAT2-deficient and WT cells at the different ratios, however, cells lacking only NFAT1 or both NFAT1 and NFAT2 showed reduced killing ability (Figure 5F). To further determine the mechanism behind this reduced cytotoxicity, we measured the expression of cytokines or lytic molecules. We found a reduction in *Prf1, Ifng*, and *Il2* mRNA levels in cells with NFAT1 deficiency (either single KO or NFAT1/2 DKO cells) (Figure S5D). Moreover, we also observed a reduction in IFN-γ, IL-2, and TNF-α cytokine production upon restimulation of these NFAT1-deficient memory-like CTLs compared to WT controls (Figures S5D–F). Thus, our data suggests that NFAT1 is mainly responsible for proper effector CTL differentiation and function, which is further strengthen upon NFAT2 deficiency.

## Discussion

NFAT family members have redundant and distinct (sometimes even opposite) roles in lymphocytes development and function. Studies performed *in vitro* have demonstrated the requirement of different NFATs in CTL cytokine production but their roles in CD8^+^ T cell differentiation has yet to be determined *in vivo* (27). In our study, we found that NFAT1 and NFAT2, despite showing similar DNA binding motif specificity and belonging to the same transcription factor family, distinctively regulate CD8^+^ T cell differentiation *in vivo*. NFAT1 is needed to generate a proper CD8^+^ T cell effector population whereas NFAT2 is required for promoting memory CTL formation, indicating distinct and even opposing roles in CD8^+^ T cell differentiation. Compound deletion of both NFAT1 and NFAT2 in CTLs resulted in a significant increase in effector memory cells (expressing high levels of both KLRG1 and CD127) suggesting a delayed transition to memory cells, and a possible additive effect upon deficiency of these two transcription factors. NFAT1/2 DKO antigen-specific CD8^+^ T cells are significantly less compared to WT counterparts at the peak of the immune response, suggesting an impaired proliferation, which is in accord with NFAT controlling cell-cycle entry and proliferation of activated T cells (40). However, CTLs lacking either NFAT1 or NFAT2 are sustained differently upon LCMV^Arm^ infection: NFAT1 deficiency led to an increased CD44^hi^ CD8^+^ population in contrast to lack of NFAT2, which resulted in reduced CD44^hi^ CD8^+^ cells compared to WT controls. Our data is in line with a recent report where expression of NFAT2 in Store-Operated Calcium Entry (SOCE)-deficient T cells restores T cell proliferation *in vivo* (40). Similarly, a previous report showed that NFAT1 represses cell proliferation whereas NFAT2 promotes cell proliferation in NIH 3T3 cells (41).

NFAT deficiency results in dysregulated expression of Tbet and Eomes, two essential transcription factors that can determine CD8^+^ T cell fate commitment. While single NFAT deficiency had an increased Eomes:Tbet ratio, cells lacking both NFAT1 and NFAT2 showed the highest Eomes:Tbet ratio. Both NFAT1 and NFAT2 have been shown to directly bind to two regulatory elements in *Tbx21* loci, one 11kb upstream and another one in the intronic region, which could account for the decrease in Tbet expression upon NFAT deficiency (37) (Figure S4). NFAT1 and NFAT2 also bind to *Eomes* regulatory elements. Other transcriptional regulators could also compete for NFAT binding and/or Eomes expression regulation. Eomes expression has been shown to be upregulated in exhausted CD8^+^ T cells during chronic infections and cancer (2); mice with dual deficiency in NFAT1 and NFAT2 are unable to clear LCMV even in an acute infection model, and similarly express the highest Eomes levels. This viral persistence has also been observed in mice with dual deficiency of STIM1 and STIM2, which are upstream of NFAT signaling pathway. Moreover, these Stim1^−/−^ Stim2^fl/fl^ CD4Cre^+^ mice also displayed similar CD8^+^ T cell differentiation defects as observed in the absence of both NFAT1 and NFAT2 (39). The viral persistence also correlates with a significant decrease in frequency of cells recognizing the immunodominant NP_396−404_ epitope, suggesting chronic antigen stimulation and exhaustion of CTLs in these mice (29, 42). The positive correlation between viral titers and KLRG1^hi^ CD127^lo^ population on day 30 again echoes chronic antigen stimulation. Given that expression of inhibitory receptors associated with exhaustion are directly regulated by NFAT family members (27), the assessment of exhaustion cannot be determined using these markers. Our phenotypic analysis in germline KO mice was also confirmed using P14 adoptive transfer and mixed bone marrow chimera experiments, demonstrating that NFAT transcription factors regulate CTL differentiation in a cell-intrinsic fashion.

NFAT1, but not NFAT2, controls effector CTL differentiation and cytotoxicity function. Our results show that cells lacking NFAT1 have an enhanced memory CTL commitment *in vivo*, and show decreased cytotoxicity against mammary carcinoma cells expressing cognate antigen. On the contrary, NFAT2-deficient cells showed increased terminal effector CTL commitment *in vivo* and normal cytotoxicity function compared to WT counterparts. Surprisingly, it has been recently reported that NFAT2 control cytotoxicity in CD8^+^ T cells in a study utilizing MHC mismatch killing of MOPC 315 plasmacytoma cells and A20J cells (37). Our *in vitro* killing assay is antigen-specific, which resembles more of CTL *in vivo* function compared to MHC mismatch-induced killing. Another report recently found a role for NFAT2 in antitumor function by controlling advanced-stage non-small cell lung cancer (NSCLC); regional NFAT2 expression correlated with tumor prognosis (43). To support their argument, Heim et al. deleted NFAT2 in both CD4^+^ and CD8^+^ T cells which resulted in severe tumor development. Given that NFAT2 is essential for supporting T cell proliferation (40), the loss of tumor control could be due to a reduction in T cell numbers. Moreover, a defect in both CD4^+^ and CD8^+^ T cell function was observed, which impedes the understanding of cell-intrinsic effects. Therefore, the differences in NFAT1 and NFAT2 deficient CTL killing capacity need further illustration *in vivo*. Additionally, the dysregulated effector and memory CD8^+^ T cell differentiation upon NFAT1 and NFAT2 deficiency could also have differential effects in the effector and memory phase. Nevertheless, our *ex vivo* and *in vitro* stimulation of NFAT deficient CD8^+^ T cells recapitulate previous findings that NFAT1 mainly regulates production of IFN-γ while other NFATs might have compensatory roles upon re-stimulation *in vitro* (27).

The early transcriptional regulation of CD8^+^ T cell differentiation that set the epigenetic tone for gene expression prior to the proliferative burst is not fully understood (44). BATF and IRF4 have also been shown to function as pioneer factors required for CTL differentiation (13, 15). However, recently Pipkin and colleagues demonstrated that Runx3 functions upstream of these factors, working as a pioneer transcription factor that induces chromatin accessibility of cis-regulatory landscapes crucial for memory CTL formation (18). NFATs are among the primary regulators downstream of TCR signaling, being activated and translocated to the nucleus within minutes of TCR stimulation (23), and are therefore expected to have profound roles during priming. This idea is consonant with the loss of chromatin accessible regions containing NFAT binding sites in Runx3 knockout CD8^+^ T cells within the first 24 h of stimulation (18). Furthermore, it has also been suggested that NFATs directly regulate IRF4 expression and form an IRF4-NFAT-BATF transcriptional circuit (45). Similarly, NFAT factors have also been reported to control expression of transcription factor HIF1a as well as metabolic genes, pathways that can also affect CD8^+^ T cell fate upon activation (40, 46–48). Thus, NFAT family members could control the transcriptional induction of several genes that can coordinately regulate CTL differentiation *in vivo*, suggesting that they could function as non-redundant pioneer transcription factors driving effector and memory CTL commitment. Future work on better understanding the role of NFAT family members can help elucidate these distinct mechanisms.

We have identified that NFAT1 and NFAT2 have opposite functions in CTL differentiation. These differential roles of transcription factors belonging to the same family have been explored. For example, Id2 is essential for generating SLEC population whereas Id3 is crucial for long-lived memory CTLs (10). IL-10-IL-21-STAT3 axis promotes CD8^+^ T cell memory differentiation and preserve cell stemness (49), while IL-12-STAT4 axis downregulates TCF1 expression to promote effector cell differentiation (50). Another example is the Egr family members, which also display opposing functions: Egr-2 and−3 play as TCR-induced negative regulators of T cell function, whereas Egr-1 promotes T cell function (51, 52). While the induction of Egr-2 and Egr-3 expression by NFAT can be independent of AP-1 (27), Egr-1 requires both NFAT and AP-1 to induce its expression in CD4^+^ T cells (53).

NFAT1 and NFAT2 could distinctly control gene expression, driving CTL differentiation through distinct mechanisms. Three possible mechanisms are: (i) the structural differences between NFATs, especially in the transactivation region (TAD) domain (54); (ii) the timing of activation, where NFAT2 short isoform expression is induced by NFAT1 activation (55); (iii) the unique binding partners of different NFATs even in different NFAT isoforms. Recent mass spectrometric analysis showed a higher number of NFAT1 unique associated proteins compared to NFAT2, suggesting these family members could distinctly regulate gene expression to induce CTL differentiation by cooperating with different transcription partners (56). The challenge of understanding the mechanism behind the distinct function of NFAT members should be further investigated during early T cell activation. How NFATs cooperate with other transcriptional regulators immediately after priming to form the chromatin landscape that eventually leads to effector and memory differentiation still remains unclear. Nonetheless, our work demonstrates the differential regulation of CD8^+^ T cell differentiation by NFAT1 and NFAT2 in acute viral infection, and provides a framework for understanding the roles of NFATs in early priming, effector and memory CD8^+^ T cell function.

## Author Contributions

TX and GM designed the experiments and wrote the article. TX performed experiments, analyzed and plotted the data. AK maintained the mouse colony and helped with *in vivo* experiments. GM supervised the project.

### Conflict of Interest Statement

The authors declare that the research was conducted in the absence of any commercial or financial relationships that could be construed as a potential conflict of interest.
