# Supplementary material for: NFAT1 and NFAT2 Differentially Regulate CTL Differentiation Upon Acute Viral Infection
Source: Front Immunol. 2019 Feb 15;10:184. doi: 10.3389/fimmu.2019.00184 (PMC6384247; doi:10.3389/fimmu.2019.00184)
Supplement: Supplementary file 2 [file Data_Sheet_1.pdf]

Supplementary Material

NFAT1 and NFAT2 differentially regulate CTL differentiation upon acute viral infection

Tianhao Xu[1], Ashleigh Keller[1], Gustavo J. Martinez[1,*]

[1]Department of Microbiology and Immunology, Chicago Medical School, Rosalind Franklin University, 3333 Green Bay Road, North Chicago, IL 60064, USA.

*Correspondence:
Dr. Gustavo Martinez
Gustavo.martinez@rosalindfranklin.edu

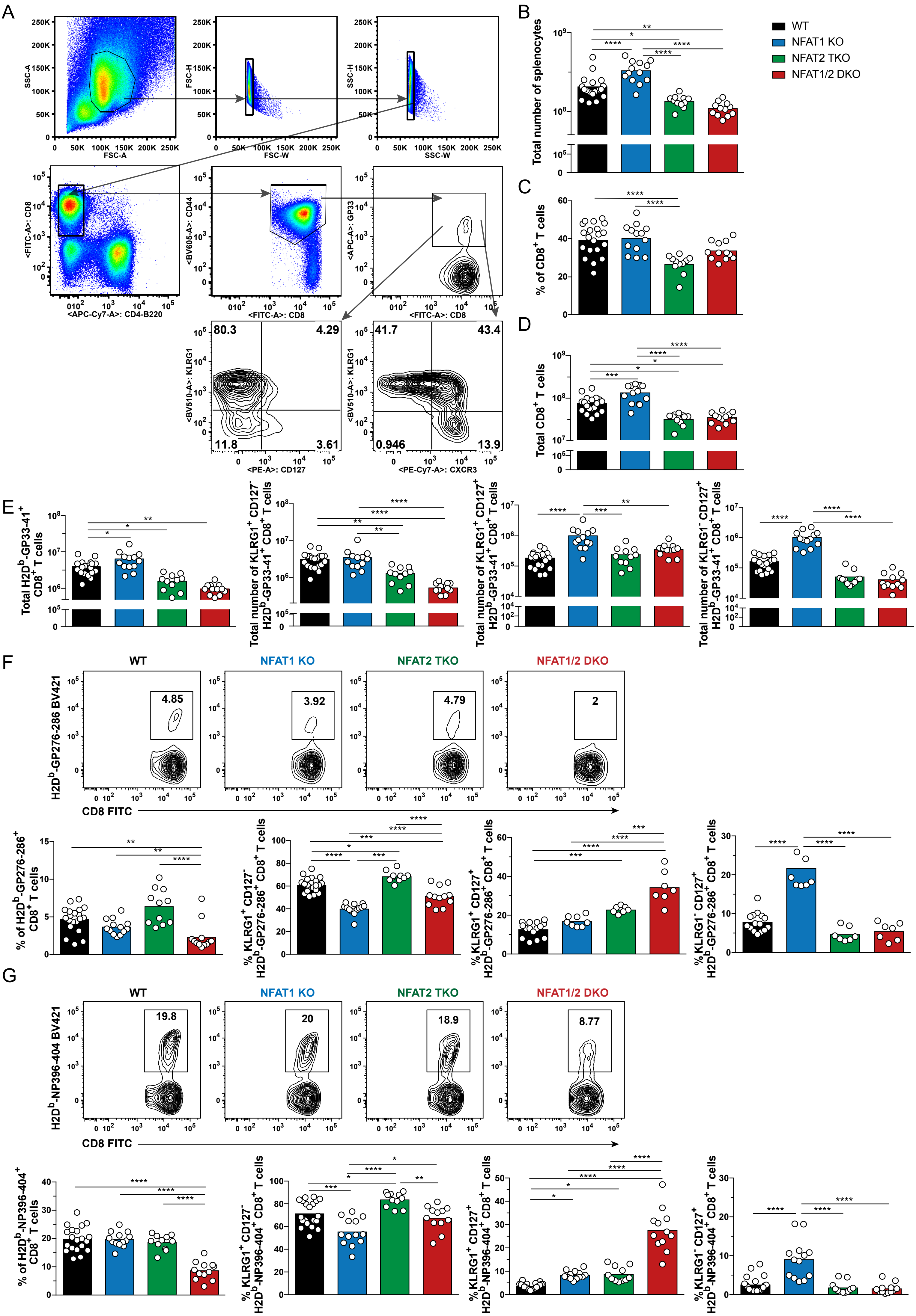
A
SSC-A
FSC-A
FSC-H
FSC-W
SSC-H
SSC-W
<FITC-A>: CD8
<APC-Cy7-A>: CD4-B220
<BV605-A>: CD44
<FITC-A>: CD8
<APC-A>: GP33
<FITC-A>: CD8
<BV510-A>: KLRG1
<PE-A>: CD127
80.3
4.29
11.8
3.61
<BV510-A>: KLRG1
<PE-Cy7-A>: CXCR3
41.7
43.4
0.946
13.9
B
Total number of splenocytes
WT
NFAT1 KO
NFAT2 TKO
NFAT1/2 DKO
C
% of CD8+ T cells
D
Total CD8+ T cells
E
Total H2D^b-GP33-41+ CD8+ T cells
Total number of KLRG1+ CD127- H2D^b-GP33-41+ CD8+ T cells
Total number of KLRG1+ CD127+ H2D^b-GP33-41+ CD8+ T cells
Total number of KLRG1- CD127+ H2D^b-GP33-41+ CD8+ T cells
F
WT
NFAT1 KO
NFAT2 TKO
NFAT1/2 DKO
H2D^b-GP276-286 BV421
CD8 FITC
4.85
3.92
4.79
2
% of H2D^b-GP276-286+ CD8+ T cells
% KLRG1+ CD127- H2D^b-GP276-286+ CD8+ T cells
% KLRG1+ CD127+ H2D^b-GP276-286+ CD8+ T cells
% KLRG1- CD127+ H2D^b-GP276-286+ CD8+ T cells
G
WT
NFAT1 KO
NFAT2 TKO
NFAT1/2 DKO
H2D^b-NP396-404 BV421
CD8 FITC
19.8
20
18.9
8.77
% of H2D^b-NP396-404+ CD8+ T cells
% KLRG1+ CD127- H2D^b-NP396-404+ CD8+ T cells
% KLRG1+ CD127+ H2D^b-NP396-404+ CD8+ T cells
% KLRG1- CD127+ H2D^b-NP396-404+ CD8+ T cells

Figure S1. Defective CTL differentiation in different antigen-specific cells upon NFATs deficiency on day 8 post LCMV Arm infection. (A) General gating strategy used in FACS analysis. **(B-D)** Total splenocytes, percentage of $CD8^+$ T cells, and total number of $CD8^+$ T cells combined form three biological replicates. **(E)** Absolute number of antigen-specific $H2D^b$-gp33-41^+ $CD8^+$ T cells and each gated KLRG1 and CD127 sub-populations form three individual experiments. **(F)** Top panel shows a representative contour plot displaying the frequency of $H2D^b$-GP276-286^+ $CD8^+$ T cells within the different mice; bottom panel shows the total cell number of $H2D^b$-GP276-286^+ $CD8^+$ T cells per spleen and frequency of KLRG1 and CD127 sub-populations within these antigen-specific cells. **(G)** Top panel shows a representative contour plot displaying the frequency $H2D^b$-NP396-404^+ $CD8^+$ T cells within the different mice; bottom panel shows the total cell number of $H2D^b$-NP396-404^+ $CD8^+$ T cells per spleen and frequency of KLRG1 and CD127 sub-populations within these antigen-specific cells. Statistical analysis was done using non-paired One-Way ANOVA followed by Tukey's multiple comparisons. *: $p \leq 0.05$, **: $p \leq 0.01$, ***: $p \leq 0.001$, ****: $p \leq 0.0001$.

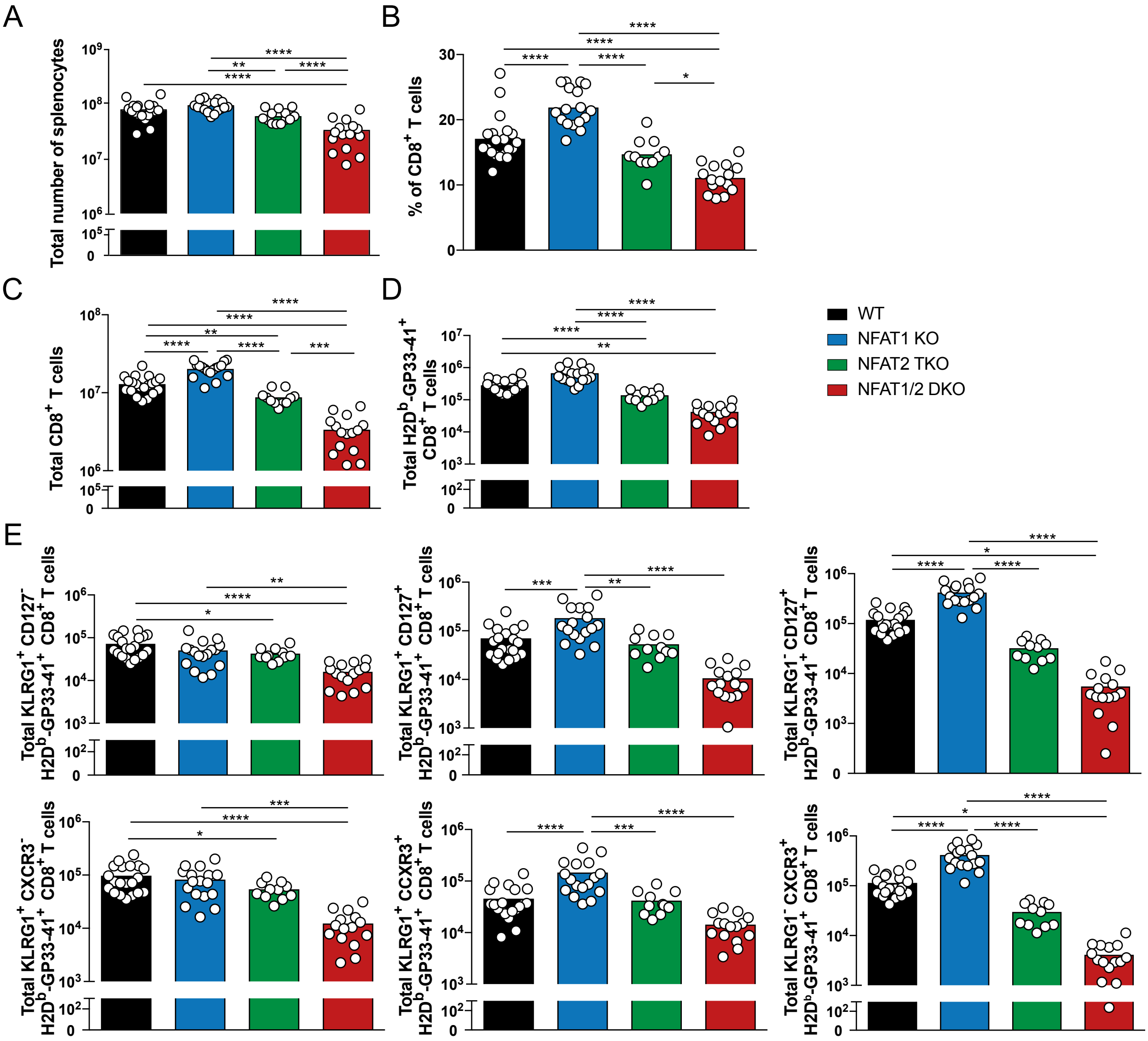

A
B
C
D
E
Total number of splenocytes
% of CD8+ T cells
Total CD8+ T cells
Total H2Db-GP33-41+ CD8+ T cells
Total KLRG1+ CD127- H2Db-GP33-41+ CD8+ T cells
Total KLRG1+ CD127+ H2Db-GP33-41+ CD8+ T cells
Total KLRG1- CD127+ H2Db-GP33-41+ CD8+ T cells
Total KLRG1+ CXCR3- H2Db-GP33-41+ CD8+ T cells
Total KLRG1+ CCXR3+ H2Db-GP33-41+ CD8+ T cells
Total KLRG1- CXCR3+ H2Db-GP33-41+ CD8+ T cells
WT
NFAT1 KO
NFAT2 TKO
NFAT1/2 DKO

Figure S2. Defective CD8$^+$ T cell differentiation upon deficiency of NFAT family members is maintained during the memory phase. Total splenocytes (**A**), percentage and total numbers of CD8$^+$ T cells (**B-C**), total number of tetramer H2D^b-GP33-41$^+$ CTLs (**D**), each gated KLRG1 and CD127 or CXCR3 sub-population within these antigen-specific cells from three individual experiments (**E**)**.** Statistical analysis was done using non-paired One-Way ANOVA followed by Tukey's multiple comparisons. *: $p \leq 0.05$, **: $p \leq 0.01$, ***: $p \leq 0.001$, ****: $p \leq 0.0001$.

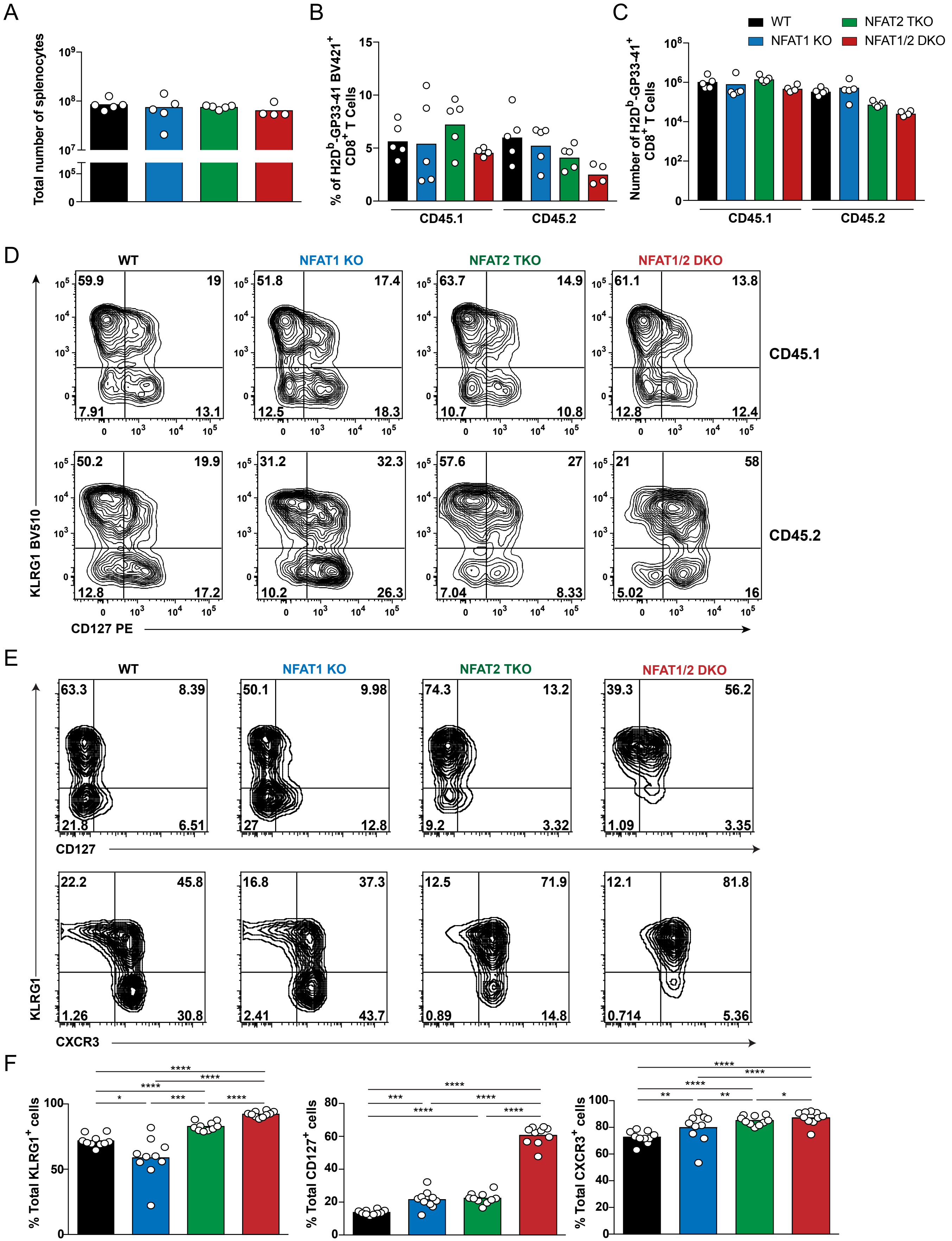
A
Total number of splenocytes
B
% of H2D^b-GP33-41 BV421^+ CD8^+ T Cells
CD45.1
CD45.2
C
Number of H2D^b-GP33-41^+ CD8^+ T Cells
WT
NFAT1 KO
NFAT2 TKO
NFAT1/2 DKO
D
WT
NFAT1 KO
NFAT2 TKO
NFAT1/2 DKO
59.9 19 7.91 13.1
51.8 17.4 12.5 18.3
63.7 14.9 10.7 10.8
61.1 13.8 12.8 12.4
CD45.1
50.2 19.9 12.8 17.2
31.2 32.3 10.2 26.3
57.6 27 7.04 8.33
21 58 5.02 16
CD45.2
KLRG1 BV510
CD127 PE
E
WT
NFAT1 KO
NFAT2 TKO
NFAT1/2 DKO
63.3 8.39 21.8 6.51
50.1 9.98 27 12.8
74.3 13.2 9.2 3.32
39.3 56.2 1.09 3.35
CD127
22.2 45.8 1.26 30.8
16.8 37.3 2.41 43.7
12.5 71.9 0.89 14.8
12.1 81.8 0.714 5.36
KLRG1
CXCR3
F
% Total KLRG1^+ cells
% Total CD127^+ cells
% Total CXCR3^+ cells

Figure S3. NFAT members regulate $CD8^+$ T cell differentiation in a cell-intrinsic manner. (**A**) Spleen cell counts. (**B-C**) Frequency and total number of $H2D^b$-GP33-41^+ $CD8^+$ T cells. (**D**) Representative contour plot displaying the expression of KLRG1 and CD127 in $H2D^b$-GP33-41^+ recipient (CD45.1) or donor (CD45.2) $CD8^+$ T cells from mix bone marrow chimera. (**E**) Representative plot showing the expression of KLRG1, CD127 and CXCR3 on adoptively-transferred P14 $CD8^+$ T cells. (**F**) The combined frequency of total $KLRG1^+$, $CD127^+$ or $CXCR3^+$ adoptively-transferred P14 $CD8^+$ T cells from two independent experiments. Non-paired One-Way ANOVA followed by Tukey's multiple comparisons were used in (**A-D**), and student's T test was used in (**F**). *: $p \leq 0.05$, **: $p \leq 0.01$, ***: $p \leq 0.001$, ****: $p \leq 0.0001$.

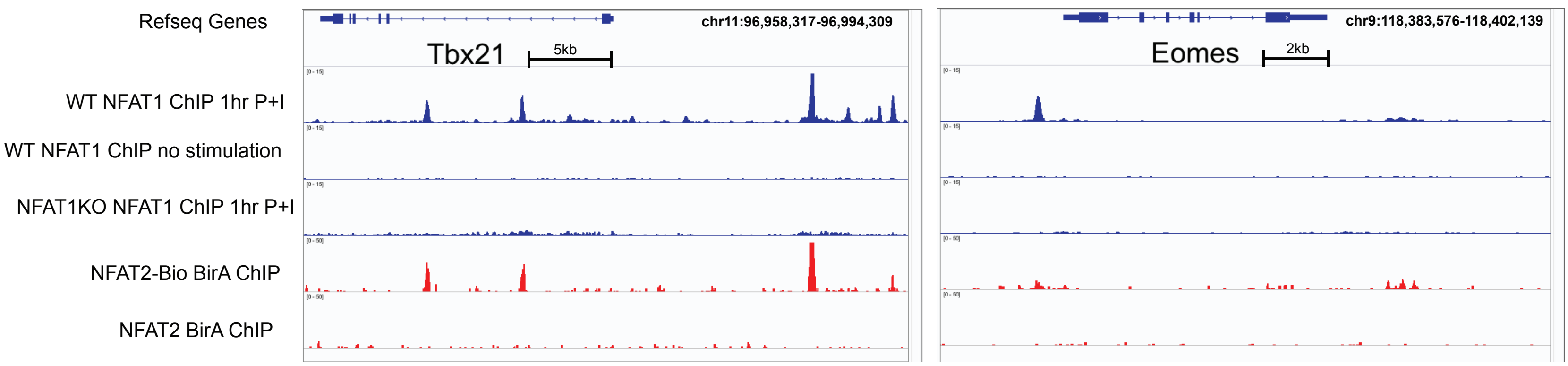
Refseq Genes
WT NFAT1 ChIP 1hr P+I
WT NFAT1 ChIP no stimulation
NFAT1KO NFAT1 ChIP 1hr P+I
NFAT2-Bio BirA ChIP
NFAT2 BirA ChIP
chr11:96,958,317-96,994,309
Tbx21
5kb
chr9:118,383,576-118,402,139
Eomes
2kb

Figure S4. NFAT1 and NFAT2 bind to Txb21 and Eomes loci. The binding of NFAT1 and biotin Tagged NFAT2 on *Tbx21* and *Eomes* loci is depicted with igv_2.4.13 using published ChIP-seq data GSE64409 and GSE98726.

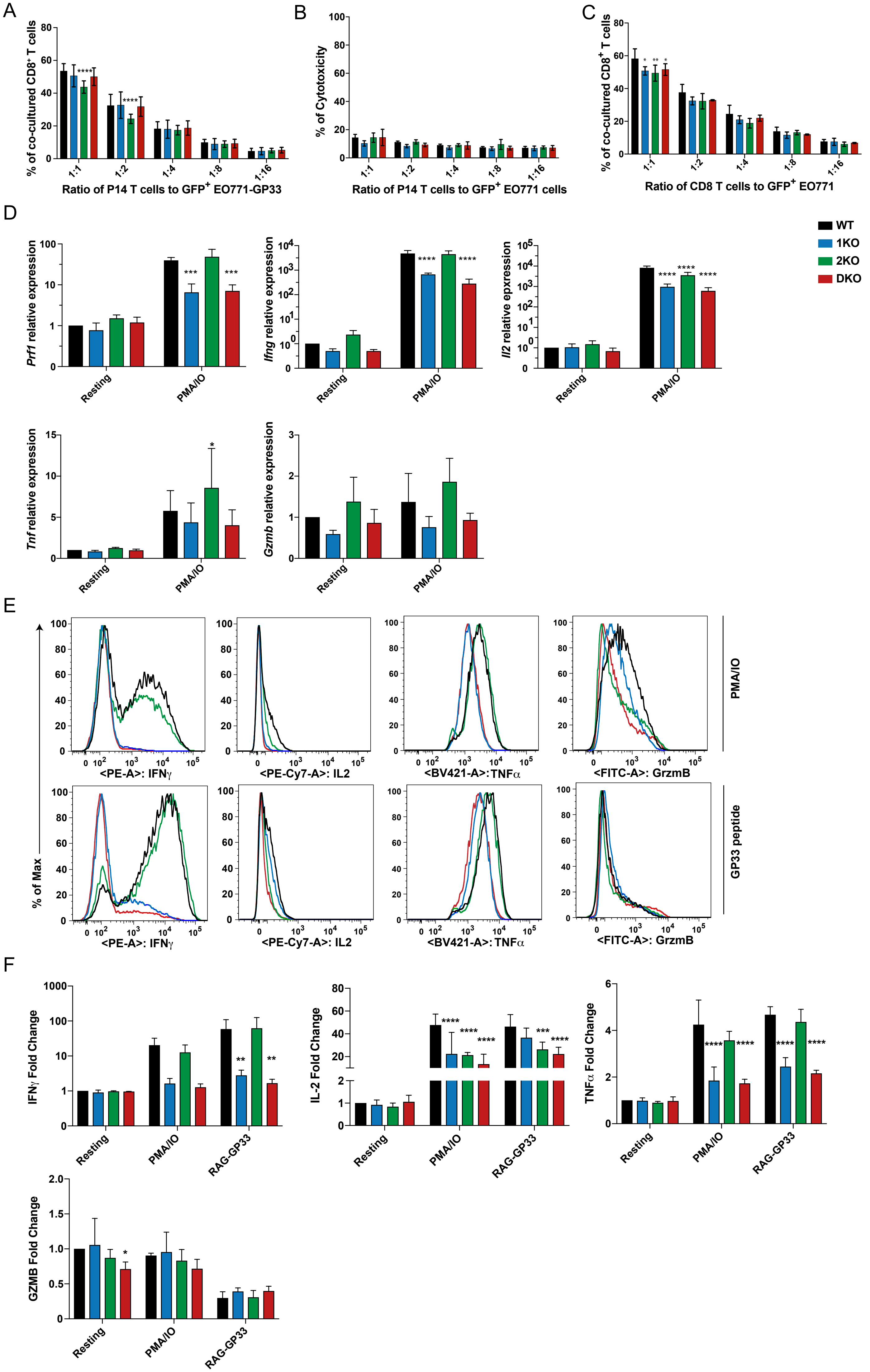

A
% of co-cultured CD8+ T cells
Ratio of P14 T cells to GFP+ EO771-GP33
1:1
1:2
1:4
1:8
1:16
B
% of Cytotoxicity
Ratio of P14 T cells to GFP+ EO771 cells
C
% of co-cultured CD8+ T cells
Ratio of CD8 T cells to GFP+ EO771
D
WT
1KO
2KO
DKO
Prf1 relative expression
Ifng relative expression
Il2 relative epxression
Tnf relative expression
Gzmb relative expression
Resting
PMA/IO
E
% of Max
<PE-A>: IFNγ
<PE-Cy7-A>: IL2
<BV421-A>:TNFα
<FITC-A>: GrzmB
PMA/IO
GP33 peptide
F
IFNγ Fold Change
IL-2 Fold Change
TNFα Fold Change
GZMB Fold Change
Resting
PMA/IO
RAG-GP33

Figure S5. Defective cytokine production and cytotoxicity upon NFAT deficiency. (A) The mean percentage of $CD8^+$ T cells incubated at different ratios with EO771 cells expressing GP33-41 from three independent experiments. **(B-C)** Mean cytotoxicity (**B**) or percentage of P14 $CD8^+$ T cells (**C**) when incubated at different ratios with parental EO771 cells without GP33-41 antigen. (**C**) Cytotoxicity was measured by the frequency of GFP^- cells within the $CD8^-$ population from three independent experiments. (**D**) Expression of effector genes in NFAT-deficient CTLs. P14 $CD8^+$ T cells used for cytotoxicity assay were restimulated with PMA and ionomycin for 4hrs, after which RNA was extracted and expression of genes determined by reat time RT-qPCR. *Rpl32* was used as control and the relative mRNA expression of different groups was determined by normalizing to WT resting condition (set as 1). (**E-F**) The representative and combined expression of IFN-γ, TNF-α, IL-2 and Granzyme B upon restimualtion of *in vitro* activated P14 $CD8^+$ T cells from the indicated genotypes. Cells were restimulated with PMA (10nM) and Ionomycin (1μM) or $Rag^{-/-}$ splenocytes primed with GP33-41 peptide (0.2ug/ml), both in the presence of Brefeldin A, and intracellular staining performed after 4h of stimulation. The mean of expression fold change was calculated by normalizing to WT resting condition from three independent experiments. Two-Way ANOVA followed by Dunnett comparisons were performed to analysis within group statistical differences. *: $p \leq 0.05$, **: $p \leq 0.01$, ***: $p \leq 0.001$, ****: $p \leq 0.0001$.
